# Toxicity Evaluation, Oxidative, and Immune Responses of Mercury on Nile Tilapia: Modulatory Role of Dietary *Nannochloropsis oculata*

**DOI:** 10.1007/s12011-023-03771-4

**Published:** 2023-07-25

**Authors:** Eman Zahran, Fatma Ahmed, Zeinab Hassan, Iman Ibrahim, Asmaa A. Khaled, Dušan Palić, Mahmoud G. El Sebaei

**Affiliations:** 1 Department of Aquatic Animal Medicine, Faculty of Veterinary Medicine, Mansoura University, Mansoura, 35516 Egypt; 2 Department of Zoology, Faculty of Science, Sohag University, Sohag, 82524 Egypt; 3 Fish Disease Department, Faculty of Veterinary Medicine, Aswan University, Aswan, 81528 Egypt; 4 Pathology Department, Faculty of Veterinary Medicine, Mansoura University, Mansoura, 35516 Egypt; 5 Animal and Fish Production Department, Faculty of Agriculture Saba Basha, Alexandria University, Alexandria, Egypt; 6 Chair for Fish Diseases and Fisheries Biology, Ludwig-Maximilians-University Munich, Munich, 80539 Germany; 7 Department of Biomedical Sciences, College of Veterinary Medicine, King Faisal University, Al-Ahsa, 31982 Saudi Arabia; 8 Department of Biochemistry and Molecular Biology, Faculty of Veterinary Medicine, Mansoura University, Mansoura, 35516 Egypt

**Keywords:** Acetylcholinesterase activity, Environmental pollutants, Mercury toxication, *Microalgae*, *Oreochromis niloticus*

## Abstract

The current study evaluated the potential ameliorative effect of a dietary immune modulator, *Nannochloropsis oculata* microalga, on the mercuric chloride (HgCl_2_)-induced toxicity of Nile tilapia. Nile tilapia (45–50 g) were fed a control diet or exposed to ¼ LC_50_ of HgCl_2_ (0.3 mg/L) and fed on a medicated feed supplemented with *N. oculata* (5% and 10% (50 or 100 g/kg dry feed)) for 21 days. Growth and somatic indices, Hg^2+^ bioaccumulation in muscles, and serum acetylcholinesterase (AChE) activity were investigated. Antioxidant and stress-related gene expression analyses were carried out in gills and intestines. Histopathological examinations of gills and intestines were performed to monitor the traits associated with Hg^2+^ toxicity or refer to detoxification. Hg^2+^ toxicity led to significant musculature bioaccumulation, inhibited AChE activity, downregulated genes related to antioxidants and stress, and elicited histopathological changes in the gills and intestine. Supplementation with *N. oculata* at 10% was able to upregulate the anti-oxidative-related genes while downregulated the stress apoptotic genes in gills and intestines compared to the unexposed group. In addition, minor to no histopathological traits were detected in the gills and intestines of the *N. oculata*-supplemented diets. Our data showed the benefit of dietary *N. oculata* in suppressing Hg^2+^ toxicity, which might support its efficacy as therapeutic/preventive agent to overcome environmental heavy metal pollution in aquatic habitats.

## Introduction

Fish environmental diseases are caused by numerous ecological contaminants, including heavy metals [1]. Aquatic contamination by heavy metals might happen from atmospheric deposition or industrial activities; therefore, their bioaccumulation was frequently reported in different aquatic ecosystems worldwide [2–4]. Several anthropogenic activities, such as fossil fuel and coal combustions or agricultural and industrial consumptions, release heavy metals causing severe contamination of the adjacent aquatic habitats. These metals are dangerous for aquatic organisms as they could persist in their environment and accumulate in their organs, negatively affecting their health, growth, meat quality, and reproduction and leading to death or extended harm to their offspring [5–7]. Furthermore, the damage of such elements extends to the human consumers of fish and seafood [8–10] or through agricultural product consumption [11].

Mercury (Hg) is one of the toxic heavy metals that spread out in aquatic habitats mainly from agricultural waste containing pesticides, in addition to occurring naturally in the atmosphere and could be deposited via weathering [6]. It is considered the third most dangerous environmentally heavy metal pollutant after arsenic and lead, found in the environment in three different forms elementary (Hg_0_), organic (methylmercury “MeHg”), and inorganic (chloride mercury “HgCl_2_”). Although MeHg is the most toxic form [12], HgCl_2_ is the most common and harmful form since it can pass across biological membranes and interact with amino acids forming organo-mercury complexes [13]. The reported Hg^2+^ toxicity for fish is in the form of brain oxidative stress, inhibition of hepatic biotransformation enzymes, genotoxicity in blood, and reproductive alterations [14–16].

Several factors can cause Hg^2+^ accumulation in fish tissues. Environmental factors such as pH of the water, dissolved organic carbon, and other biological factors including fish age, size, foraging habitat, and primary productivity can all influence bioaccumulation [6, 17]. Low primary productivity leads to high Hg^2+^ concentrations because of the algal bio-dilution, which increases the accumulated MeHg levels [6]. Additionally, Hg^2+^ concentrations increased with fish size, and higher concentrations were reported in the bottom (demersal) than in the surface (pelagic) fish and concerning percent floodable area rather than the hydromorphic soils, which did not influence fish Hg level [6].

The inorganic mercury compounds suppress the antioxidant defense system in host bodies and induce apoptosis. Chelating agents are used in medicine to treat mercury toxicity by forming chelation compounds with toxic metal ions that are easily excreted through the excretory system [13]. On the other hand, natural antioxidants are preferable because drugs are ineffective at repairing tissue damage and may cause toxic side effects. Using the biological systems for mercury absorption reported augmentation of Hg^2+^ accumulation in the aquatic organisms’ organs [18].

Several natural antioxidant products have recently been used as potential non-toxic therapies against heavy metals and microalgae toxicity. Microalgae are essential metabolites for food and medicine [19] and have gained high importance in the aquaculture industry [20] since they were frequently used as feed and food additives, in addition to being live feed in aquaculture [21]. Furthermore, surfaces of algal cells possess several kinds of functional groups responsible for heavy metals’ chelation from contaminated water [22]. Microalgae, including *N. oculata*, rapidly respond to changes in element availability; therefore, they can be used to evaluate contaminated areas and predict the ecological effects of pollution.

The current study focuses on Hg^2+^ toxicity because it is a ubiquitous heavy metal; MeHg and HgCl_2_ naturally occur in the water and cause ultimate public health problems [23]. Besides, tilapia fish was selected as a model because it shows high resistance against water pollution during environmental toxicological studies [24, 25]. In line with our previous findings [26], the effects of mercury exposure have been studied extensively. Still, researchers have mostly focused on the cellular level, paying less attention to how exposure affects growth performances or gene expressions. Therefore, the primary goals of the present study were to examine the underlying molecular and genetic mechanisms and score analysis of the histopathological lesions. Further, the potential of the microalgae *N. oculata* as a natural protectant for HgCl_2_ pollutants was emphasized.

## Materials and Methods

### Diet Formulation

Ready-to-use *N. oculata* dry powder was purchased from the National Research Center, Cairo, Egypt. *N. oculata*-supplemented diets were prepared, as described in our previous study [26]. Two medicated diets were formulated by supplementing Nile tilapia basal feed with *N. oculata* powder at two different doses: 50 g/kg dry feed (i.e., 5% *N. oculata*) or 100 g/kg dry feed (i.e., 10% *N. oculata*). The dried pellets were placed in a plastic bag and stored at 4 °C until feeding. The composition of the medicated diets is presented in Table 1.

**Table 1:** Ingredients of basal and experimental diets (g ingredient/kg diet)

Ingredients	G_0_	G_N5+Hg_	G_N10+Hg_
Yellow corn	126	146	133
Soybean meal	203	200	190
Fish meal	200	160	150
Corn gluten	10	30	0
Gelatine	20	20	20
Sunflower oil	30	35	45
Wheat bran	400	350	350
Minerals and vitamins premix	5	5	5
Salt	3	3	3
Dicalcium phosphate	1	1	1
Methionine	2	3.2	3.2
Algae (*N*. *oculata*)	0	50	100
Proximate analysis (% dry matter basis)			
Crude Protein*	32.24	32.7	32.39
Lipid*	6.5	6.00	5.89
Ca*	1.1	0.96	0.90
P*	0.50	0.47	0.45
DE (Digestable Energy)** (kcal/kg)	3023	3025	3000

^a^Trace minerals & vitamins premixes were prepared to cover the micro minerals &vitamins levels for tilapia fish as recommended by NRC [92]. Vitamins premix (IU or mg/kg diet); vit. A 5000, Vit.D3 1000, vit. E 20, vit. k3 2, vit. B12, vit. B25, vit. B6 1.5, vit. B12 0.02, Pantothenic acid 10, Folic acid 1, Biotin 0.15, Niacin 30. Mineral mixture (mg/kg diet); Fe 40, Mn 80, Cu 4, Zn 50, I 0.5, Co 0.2 & Se 0.2. *N*= *N. oculata* (*Nannochloropsis oculata*), G_0_= control, G_N5+Hg_= group supplemented by 5% *N*. *oculata* and exposed to HgCl_2_, and G_N10+Hg_= group supplemented by 10% *N. oculata* and exposed to HgCl_2_

^*^Analyzed

^**^Calculated value

### Fish Maintenance and Experimental Conditions

The current study was conducted at the experimental facility of the Aquatic Animal Medicine (AAM) Laboratory, Faculty of Veterinary Medicine, Mansura University, Mansoura, Egypt. Procedures for animal care and management were approved by the Ethical Research Committee of Mansoura University, Code number: MU-ACUC (VM.R.23.01.42).

Nile tilapia (*O. niloticus*, average total body weight 45–50 g, and length 14–15 cm) were obtained from a certified private fish farm at Kafr El Sheik Governorate. Fish were acclimated for two weeks to the de-chlorinated tap water in 500 L capacity fiberglass tanks supplied with adequate aeration (6.5–7.8 mg/L dissolved oxygen) and pH (7.1–7.3), and the water temperature was maintained at 24 ± 2 °C throughout the experiment. Fish were fed to satiation using commercial feed (Uccma feed, Egypt; crude protein, 32%; crude lipid, 6.2%; crude fiber, 5.7%) and were exposed to a 12 h light–dark cycle. During the acclimation period, fish were frequently checked, according to EJ Noga [27], and only healthy fish were chosen in terms of general appearance and activity level. Daily water parameters were monitored throughout the trial using water quality test kits (Aquarium Pharmaceuticals, Inc.) for each tank. Up to 50% of all aquaria’s water was siphoned and replaced twice weekly to maintain water quality parameters.

### Fish Exposure to Mercury

The fish were exposed to a sub-lethal concentration of 0.3 mg/L HgC1_2_ for up to 21 days. This concentration equates to 1/4th of the median lethal concentration of 1.21 mg/L based on a 96-h toxicological assay in Nile tilapia [26]. To summarize, a new daily stock solution of 1000 mg/L mercury was made by dissolving the calculated amount of HgCl_2_ in one liter of double-distilled water. The concentration in mg/L was then determined by adding a known volume of the stock solution to the glass aquaria. Fish not exposed to HgCl_2_ were kept in separate tanks under identical conditions.

### Experimental Design

After acclimation, 120 selected healthy Nile tilapia fish were used for our experiment, which extended for 21 days. Twelve 80 L fiberglass indoor tanks with a primary stocking density of 10 fish per tank were randomly assigned into four groups in triplicate (i.e., 30 fish/group). Fish grouping conditions were kept considering similar “tank effects” between the replicate tanks. On the first day of the experiment, all groups were exposed for 21 days to the quarter value of the determined Hg^2+^ LC_50_ (i.e., 0.3 mg/L HgCl_2_), except one unexposed control group. Two groups received basal diets throughout the experiment, including the control group (*G*_0_) and one of the Hg^2+^ exposed groups (*G*_Hg_). The other two groups were exposed to the same concentration of Hg^2+^ and simultaneously received medicated diets; a 5% *N. oculata*-supplemented diet was introduced to the third group (*G*_N5+Hg_), and a 10% *N. oculata*-supplemented diet was introduced to the fourth group (*G*_N10+Hg_). Throughout the experiment, fish were fed twice daily at 2% of their total body weight/day [28]. The leftover fish waste or feed was continuously siphoned from tanks, keeping constant clean water in all tanks. Up to 80% of the water in aquaria used to be replaced daily by a static-renewal system keeping the Hg^2+^ exposure state by adding a new daily stock solution of HgCl_2_ (0.3 mg/L) [29].

### Fish Sampling and Tissue Collection

Two fish were sampled from each aquarium tank (6 fish/group) after 21 days of feeding. Sampled fish were euthanized with an overdose of MS222 (Argent) at 200 mg/L tricaine + 400 mg/L sodium bicarbonate. Each sampled fish was weighed and measured; then, the blood samples were withdrawn from their caudal vessels using a 23-gage needle. The collected blood was kept in an inclined position for 20 min at room temperature and then centrifuged at 1700 × g for 10 min for serum separation and stored at − 20 °C until analysis. Fish were dissected immediately after euthanasia, and the muscles, gills, and intestine were excised. The muscles were immediately processed for the estimation of Hg^2+^ bioaccumulation. The gills and intestines were cut into small portions. Some portions were placed in RNA Later® (Qiagen) and kept overnight at 4 °C and then stored at − 80 °C until further use for the gene expression analysis. Other portions were fixed in 10% neutral buffer formalin for the processing of the histopathological examination.

### Fish Growth and Somatic Indices

Fish initial and final weight and length were estimated for each sampled fish to follow up on the fish growth performance. The condition factor (*K*-factor) was calculated according to the following formula: *K*-factor = [(fish weight) (g)/ (fish length (cm))^3^] × 100. In addition, the liver was excised immediately upon dissection and weighed to determine the HSI according to the following formula: HSI = (liver weight (g)/ body weight (g)) × 100.

### Total Mercury Content in Nile Tilapia Musculature

Digested samples consisted of 1 g of fish musculature placed in 5 mL of 65% nitric acid (HNO_3_) in a water bath maintained at 100 °C until a clear liquid was obtained [30]. After cooling, the fluid was filtered through Whatman filter paper (No. 42, pore size 2.5 m). Following dilution in deionized water to a final volume of 25 mL, the Hg concentration was determined using an atomic absorption spectrophotometer (AAS- Perkin Elmer Analyst 100) fitted with an MHS-10 mercury/hydride system.

### Serum Acetylcholinesterase (AChE) Activity

Acetylcholinesterase activity was estimated to evaluate organic poisoning as a biological indicator of intoxication [31]. The collected serum cholinesterase was measured following the manufacturer’s protocol of the Cobas c pack reagents using COBAS INTEGRA 400 plus analyzer Roche Diagnostics. Briefly, serum cholinesterase enzymatic activity was measured by continuous colorimetric kinetic methodology. It is based on a kinetic test by the method of butyrylthiocholine. The color decrease was measured in wavelength between 405 and 415 nm. The results were expressed in international units per liter (U/L).

### Gene Expression Analysis

Two antioxidant enzymes-relevant genes, glutathione-S-reductase (GSR) and glutathione peroxidase (GPx) genes, and two stress proteins-relevant encoding genes, heat-shock protein (HSP) and CRISPR-encoded protein (caspase 3, CAS) genes, were targeted for our study. The RNA was extracted from intestinal and gill tissues using RNAlater (Invitrogen; Thermo Fisher Scientific, Inc.), and cDNA synthesis was performed using TOPscript™ RT DryMIX (dT18/dN6 plus). Quantitative real-time PCR (qPCR) was conducted according to the manufacturer’s instructions in real-time PCR system (The Azure Biosystems Cielo™ qPCR Systems, USA) using TOPreal™ qPCR 2X PreMIX (SYBR Green with low ROX). The PCR cycling conditions included a primary denaturation at 95 °C for 15 min followed by 40 series of denaturation at 95 °C for 30 s, annealing at 60 °C for 60 s, and extension at 72 °C for 60 s. The oligonucleotide-specific primers were previously published; GSR, GPx, HSP, β-actin [32, 33], and CAS [34]. A standard curve for each gene was established to quantify the expression levels. The expression levels of each gene were calculated as arbitrary units normalized to that of β-actin. Relative expressions were calculated by comparing the average expression level of the experimental group with that of the corresponding control group.

### Histopathological Examination and Scoring System

The intestine and gills were dissected from the Nile tilapia and then collected and fixed in 10% neutral buffered formalin for 24 h. The dissected organs were placed in tissue cassettes, processed, and embedded in paraffin wax. A microtome (Leica RM2125 RTS, Germany) sliced embedded samples at 5 µm. The slices were stained using hematoxylin and eosin [35]. The stained slides were examined under a light microscope (Olympus CX 31 microscope). Additionally, the positively stained goblet cells were counted in intestinal sections of different experimental groups. The numbers were counted in four fields of view at 400 × magnifications and expressed as the mean goblet cell number per 6 fields of view. In this experiment, the lesions were analyzed in four fields of view at magnification 400 × for the gills and intestine section of three fish (3 sections per slide for each fish) in 12 fields of view per fish. Basic histopathological scoring parameters for lesions have been conducted as in Tables 2 and 3. The scale value of each lesion was determined for each fish (none: 0, mild: 1, moderate: 2, and severe: 3). The average of all scores was considered per fish [36].

**Table 2:** Basic histopathological scoring parameters for gills lesion in Nile tilapia

Score	Lamellar fusion	Lamellar degeneration and necrosis	Inflammation
0	None	None	None
1	1–4 field out of 9 field exhibited lamellar fusion	1–2 field /9 field displayed degenerative changes	1–2 field out of 9 field had inflammation
2	5–8 field/9 field exhibited lamellar fusion	3–6 field/9 field displayed degeneration with multifocal lamellar sloughing	3–4 field/9 field displayed inflammation
3	9–9/9 field displayed lamellar fusion	7–9 field/9 field displayed diffuse lamellar sloughing	5–7 field/9 field displayed inflammation

**Table 3:** Basic histopathological scoring parameters for intestinal lesion in Nile tilapia

Score	Intestinal degeneration and necrosis	Inflammation	Vascular changes
0	None	None	None
1	1–2 field /9 field exhibited degenerative changes	1–2 field out of 9 field had inflammation	1–2 field out of 9 field had occasional submucosal edema with rare hemorrhage
2	3–5 field/9 field exhibited degeneration (vacuolation)	3–4 field/9 field showed inflammation	3–4 field/9 field had minimal to mild edema with rare hemorrhage
3	6–9 field/9 field exhibited diffuse intestinal vacuolation	5–7 field/9 field showed inflammation	5–6 field/9 field had extensive edema with few hemorrhage

### Statistical Analysis

Data were first subjected to normality and homogeneity checks using Kolmogorov–Smirnov and Levene’s tests. All data, excluding the histopathological scoring, were subjected to a one-way analysis of variance (ANOVA) followed by a Tukey post hoc test to compare means between groups using GraphPad Prism v 8.4.2 (GraphPad Software, Inc., USA). Normalized individual fold change values were anchored to the lowest value recorded in each data set and then Log2 transformed, as described previously [37]. Nonparametric Kruskal–Wallis and Mann–Whitney *U* tests were used to analyze the histopathological scoring data among treatment groups and between two group comparisons. One-way ANOVA using post hoc Tukey’s multiple range tests was used to compare the mean values among experimental groups, including goblet cell numbers. All data were expressed as mean ± standard deviation (SD). Differences were considered statistically significant when *p* < 0.05 (*), *p* < 0.01 (**), *p* < 0.001 (***), and *p* < 0.0001 (****).

## Results

### Fish Growth Performance

Dietary *N. oculata* for 21 days did not significantly affect the fish growth and somatic indices, where no statistical changes were observed in the final body weight between groups. In addition, no apparent influence was observed on the *K*-factor and HSI of the exposed fish compared to each other or compared to the control unexposed fish (Table 4).

**Table 4:** The effects of HgCl_2_ exposure and diets on growth performances in Nile tilapia

Treatment group	Parameters		
	FBW (gm)	*K*-factor	HSI
Non-exposed, non-supplemented (*G*_0_)	41.20 ± 3.08	1.49 ± 0.026	2.982 ± 0.42
Hg-exposed, non-supplemented (*G*_Hg_)	50.73 ± 2.32	1.70 ± 0.055	2.9 ± 0.42
Hg-exposed, 5% *N. oculata*-supplemented (*G*_N5+Hg_)	54.8 ± 3.68	1.71 ± 0.082	2.892 ± 0.43
Hg-exposed, 10% *N. oculata*-supplemented (*G*_N10+Hg_)	49.00 ± 4.48	1.70 ± 0.061	2.692 ± 0.45

The fish were fed with the control diet (*G*_0_) or exposed to 0.3 ppm HgCl_2_ and fed with the control diet (*G*_hg_), or with diets containing 5% *Nannochloropsis oculata* (*N. oculata*) (*G*_N5+Hg_) or 10% *N. oculata* (*G*_N10+Hg_) for 21 days. All data are expressed as mean ± SD (*n* = 6/group). Different superscript letters or none on the mean values in a column indicate statistically different data (*p* < 0.05) or insignificance. (FBW) final body weight, (K-factor) condition factor = [(fish weight) (g)/ (total fish length (cm))^3^] × 100, and (HSI) hepatosomatic index = (liver weight (g)/ body weight (g)) × 100

### Bioaccumulation in Fish Muscles

**Fig. 1:**
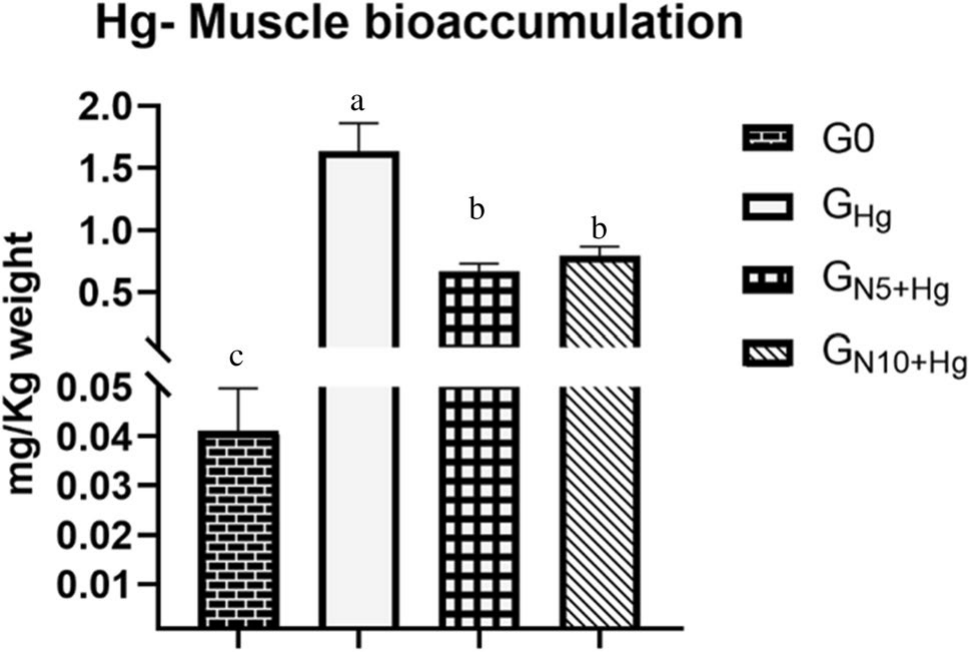
The effects of HgCl_2_ exposure and diets on Hg^2+^ accumulation in Nile tilapia. The fish were fed with the control diet (*G*_0_) or exposed to 0.3 ppm HgCl_2_ and fed with the control diet (*G*_Hg_), or with diets containing 5% *N. oculata* (*G*_N5+Hg_) or 10% *N. oculata* (*G*_N10+Hg_) for 21 days. Data are expressed as the mean ± SEM of six fish. Values with a different letter superscript are significantly different between groups. Significant levels (*p* < 0.05, 0.01, and 0.001), as determined by one-way ANOVA

Hg^2+^ accumulated in the exposed, non-supplemented tilapia muscles up to 1.5 mg/kg of their body weight. Dietary *N. oculata* decreased the Hg^2+^ content to approximately a third of its exposed group level as *N. oculata* 5%, and 10% supplementing doses decreased the accumulated amount to 0.5 and 0.8 mg/kg, respectively. Notably, small traces (0.04 mg/kg) were recorded in the muscles of the control unexposed, untreated fish (Fig. 1).

### Acetylcholinesterase (AChE) Activity

**Fig. 2:**
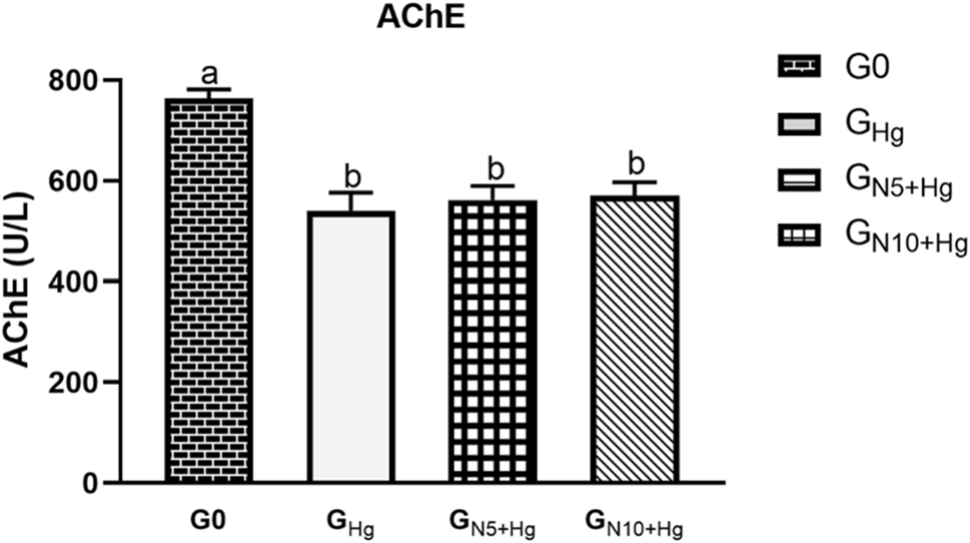
The effects of HgCl_2_ exposure and diets on serum acetylcholinesterase (AChE) in Nile tilapia. The fish were fed with the control diet (*G*_0_) or exposed to 0.3 ppm HgCl_2_ and fed with the control diet (*G*_Hg_), or with diets containing 5% *N. oculata* (*G*_N5+Hg_) or 10% *N. oculata* (*G*_N10+Hg_) for 21 days. Data are expressed as the mean ± SEM of six fish. Values with a different letter superscript are significantly different between groups. Significant levels (*p* < 0.05, 0.01, and 0.001), as determined by one-way ANOVA

A significant decrease was recorded in the AChE activity of all the exposed fish compared to the control, regardless of *N. oculata* dietary supplementation. Notably, *N. oculata* supplementation was able to augment the AChE levels, and the *G*_N10+Hg_ recorded higher augmentation than the *G*_N5+Hg_; however, it is of no significance (Fig. 2).

### Relative Expression of the Antioxidant, Stress, and Apoptotic-Relevant Genes

**Fig. 3:**
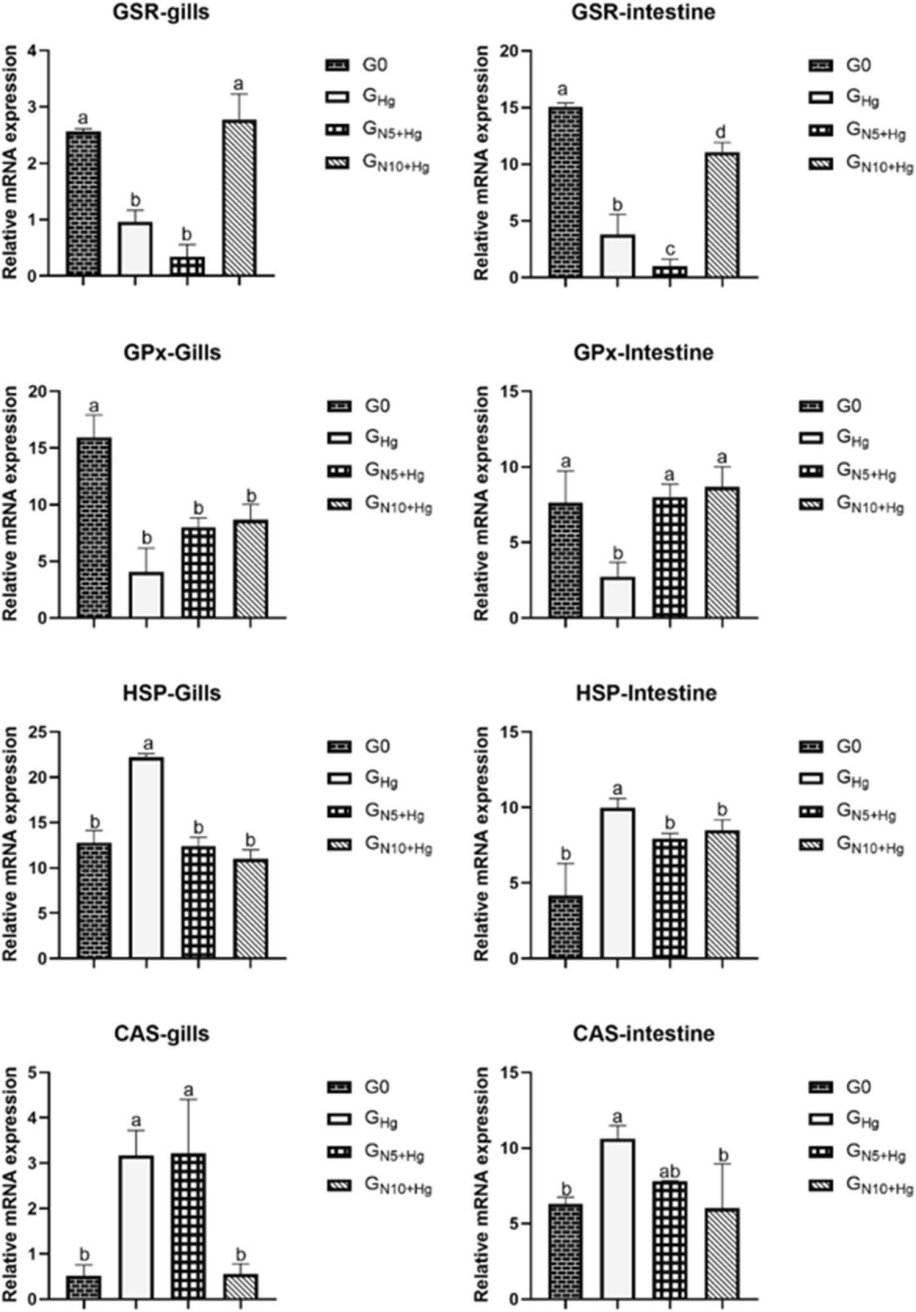
The effects of HgCl_2_ exposure and diets on the expression of genes related to antioxidant (glutathione-S-reductase (GSR) and glutathione peroxidase (GPx)), stress (heat-shock protein (HSP 70)), and apoptosis (CRISPR-encoded protein (caspase 3, CAS)) in Nile tilapia. The fish were fed with the control diet (*G*_0_) or exposed to 0.3 ppm HgCl_2_ and fed with the control diet (*G*_Hg_), or with diets containing 5% *N. oculata* (GN5 + Hg) or 10% *N. oculata* (*G*_N10+Hg_) for 21 days. Data are expressed as the mean ± SEM of six fish. Values with a different letter superscript are significantly different between groups. Significant levels (*p* < 0.05, 0.01, and 0.001), as determined by one-way ANOVA

Gene expression analysis in gills and intestine tissues is presented in Fig. 3. In our findings, Hg^2+^ exposure significantly downregulated the antioxidant enzymes related genes in gills and intestine (GSR, *p* < 0.001), (GPx *p* = 0.001, *p* = 0.038, in gills and intestine, respectively), while upregulated the stress-related genes, CAS (*p* = 0.006, *p* = 0.037, in gills and intestine, respectively), and HSP (*p* < 0.001, *p* = 0.008, in gills and intestine, respectively) compared to the unexposed control group (G0).

Our results revealed that 10% dietary *N. oculata* revealed a significant elevation in the GSR gene expression of both gills and intestines compared to the other groups (*G*_Hg_ and *G*_N5+Hg_) (*p* < 0.001). Notably, this elevation was high in gills and recorded a value close to and not significantly different (*p* > 0.05) than that of the G0 group. Similarly, 5% and 10% dietary *N. oculata* elevated the expression of the GPx gene in both gills and intestines compared to *G*_Hg_. This elevation was insignificant in the gills of *G*_N5+Hg_ and *G*_N10+Hg_ (*p* = 0.127 and *p* = 0.082, respectively). At the same time, it was significant in the intestine of *G*_N5+Hg_ and *G*_N10+Hg_ (*p* = 0.028 and *p* = 0.017, respectively), and both recorded values close to the G0 group without significant difference among them.

As for the immune-relevant genes, a significant decrease in the relative expression of the HSP-encoding gene in both gills and intestine of *G*_N5+Hg_ (*p* < 0.001 and *p* < 0.05, respectively) and *G*_N10+Hg_ (*p* < 0.001 and *p* = 0.031, respectively) compared to *G*_Hg_ group. No significant differences (*p* > 0.05) were recorded among the G0, *G*_N5+Hg_, and *G*_N10+Hg_ groups regarding the relative expression of the HSP gene. On the other hand, 10% *N. oculata*-supplemented diets significantly downregulated the relative expression of the CAS-encoding gene in both the intestine and the gills compared with the *G*_Hg_ and *G*_N5+Hg_ groups (*p* = 0.006 and *p* = 0.027 for gills and intestine, respectively). Notably, in the intestine, no significant differences (*p* = 0.53) were recorded between *G*_N5+Hg_ and *G*_N10+Hg_ and with the G0 group in the relative expression of the CAS gene (*p* = 0.659 and *p* = 0.995, respectively).

### Histopathological and Scoring Results

**Fig. 4:**
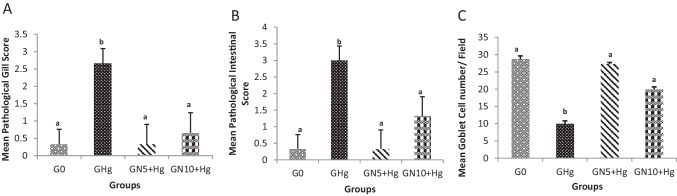
The effects of HgCl_2_ exposure and diets on pathological scores among the groups in gills (A), intestines (B), and goblet cells (C) in Nile tilapia. The fish were fed with the control diet (*G*_0_) or exposed to 0.3 ppm HgCl_2_ and fed with the control diet (*G*_Hg_), or with diets containing 5% *N. oculata* (GN5 + Hg) or 10% *N. oculata* (*G*_N10+Hg_) for 21 days. Data are expressed as the mean ± SEM of three fish. Values with a different letter superscript are significantly different between groups. Significant levels (*p* < 0.05, 0.01, and 0.001), as determined by Kruskal–Wallis and Mann–Whitney *U* tests

**Fig. 5:**
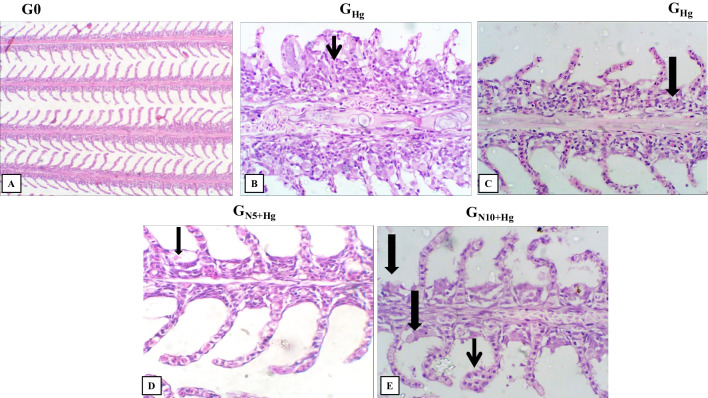
Histopathology of HgCl_2_ exposure in the gills of Nile tilapia. The fish were fed with the control diet (*G*_0_) or exposed to 0.3 ppm HgCl_2_ and fed with the control diet (*G*_Hg_), or with diets containing 5% *N. oculata* (GN5 + Hg) or 10% *N. oculata* (*G*_N10+Hg_) for 21 days. (A) control gills (G0) show a normal histological appearance of primary and secondary lamellae. (B) *G*_Hg_ showing extensive lamellar thickening by severe hyperplasia (thin arrow) with stunted, fused, or complete lack of secondary lamellae. (C) *G*_Hg_ showing lamellar branchitis with moderate numbers of inflammatory cells, predominantly lymphocytes (thick arrow). (D) *G*_N5+Hg_ shows gills apparently normal with occasional primary lamellar degeneration (thick arrow). (E) *G*_N10+Hg_ shows focal tip lamellar hypertrophy (thin arrow) with mild lamellar sloughing (thick arrows). Image magnification = 400 ×

Histopathological examination of the intestine and gills was investigated to confirm the protective effect of *N. oculata* against mercury toxicity. The histopathological score was performed for the intestine and gills (Fig. 4A–C). A significant difference in pathological effect on gills and intestine were observed between treatment groups with *p* = 0.001 (Figs. 4A and 5). The pathological effect of *G*_Hg_ on gills and intestines was significantly evident compared to the control group (*p* = 0.050 and *p* = 0.034, respectively). However, a significant reduction of the histopathological effect was detected in *N. oculata* groups compared to *G*_Hg_ with insignificant difference compared to the control group (*p* = 1.000, in the intestine and gills of *G*_N5+Hg_) and (*p* = 0.099, in the intestine, *p* = 0.453 in the gills of *G*_N10+Hg_). The gills of the control group are showing the normal histological appearance of primary and secondary lamellae (Fig. 5A). Diffuse lamellar fusion and lymphocytic bronchitis were detected in *G*_Hg_ group (Fig. 5B and C). However, few pathological findings were seen in *G*_N5+Hg_ and *G*_N10+Hg_ groups (Fig. 5D and E).

**Fig. 6:**
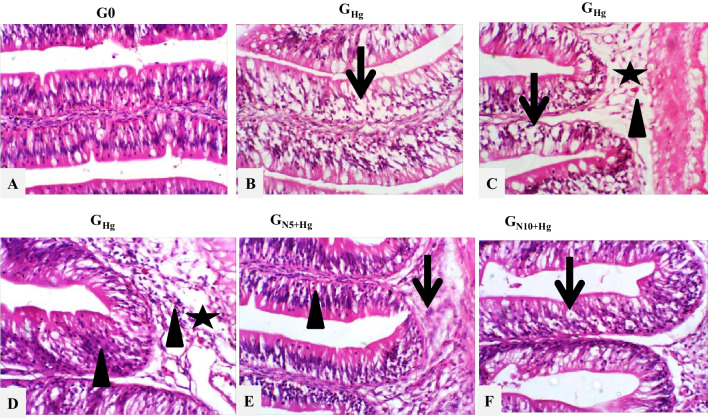
Histopathology of HgCl_2_ exposure in the intestine of Nile tilapia. The fish were fed with the control diet (*G*_0_) or exposed to 0.3 ppm HgCl_2_ and fed with the control diet (*G*_Hg_), or with diets containing 5% *N. oculata* (GN5 + Hg) or 10% *N. oculata* (*G*_N10+Hg_) for 21 days. (A) *G*_0_ intestine shows normal histological appearance. (B) *G*_Hg_ shows diffuse, extensive intestinal swollen with multiple vacuolation (thin arrow). (C) *G*_Hg_ shows showing severe intestinal vacuolation (thin arrow) extensive submucosal edema (star) admixed with few eosinophilic granular cells (arrowhead). (D) *G*_Hg_ shows extensive enteritis represented by lymphocytic infiltrations of enterocytes and extended in the submucosal layer (arrow heads), which is mixed with edema and few RBCs (star). (E) *G*_N5+Hg_ shows minimal submucosal leukocytic aggregations (thin arrow) with few lymphocytic and eosinophilic granular cells invading mucosal layer (arrowhead). (F) *G*_N10+Hg_ shows diffuse moderate enterocyte vacuolation with few submucosal lymphocytic aggregations (thin arrow). Image magnification = 400 ×

The intestine of the control group showed a normal histological appearance of the intestinal mucosa with scattered goblet cells (Fig. 6A). Meanwhile, extensive intestinal degenerative changes represented by diffuse, severe vacuolation were detected in *G*_Hg_ group (Fig. 6B). Moderate to severe enteritis were also seen characterized by moderate mucosal lymphocytic infiltrations extended to submucosal layers beside diffuse, mild to severe submucosal edema admixed with lymphocytes, and few eosinophilic granular cells (Fig. 6C and D). In contrast, the normal histological appearance of the intestine was partially restored, with insignificant differences in goblet cell numbers of *G*_N5+Hg_ and *G*_N10+Hg_ groups (Fig. 6E and F).

**Fig. 7:**
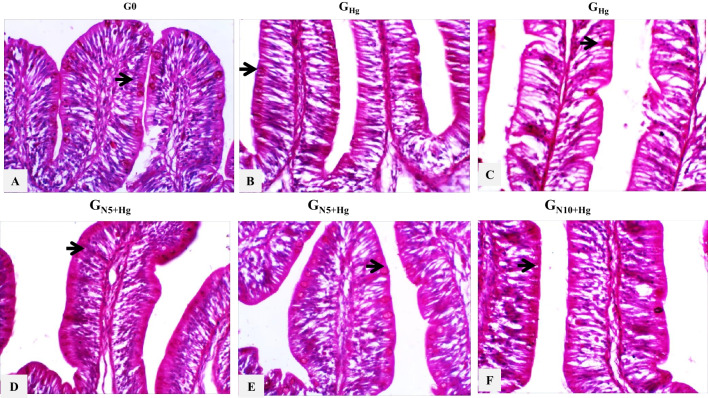
Histopathology of HgCl_2_ exposure in the intestinal goblet cells of Nile tilapia. The fish were fed with the control diet (*G*_0_) or exposed to 0.3 ppm HgCl_2_ and fed with the control diet (*G*_Hg_), or with diets containing 5% *N. oculata* (GN5 + Hg) or 10% *N. oculata* (*G*_N10+Hg_) for 21 days. (A) *G*_0_ shows normal expression of goblet cells. (B) *G*_Hg_ shows low to a mild expression of goblet cells. (C) *G*_N5+Hg_ shows similar goblet cells expression as the control. (D) *G*_N10+Hg_ shows a moderate expression of goblet cells. Thin arrow = positive PAS cells. Image magnification = 400 ×

The differential goblet cell numbers of intestinal sections were quantified between different treatment groups. There was a significant difference between groups (*p* < 0.000). Goblet cell number was significantly lower in the *G*_Hg_ group than in G0 (*p* < 0.000). In contrast, there is no significant difference between *G*_N5+Hg_ and *G*_N10+Hg_ compared to the G0 group (*p* < 0.991 and 0.070) (Figs. 4C and 7).

## Discussion

### Mercury (Hg^2+^) Toxicity

Heavy metals are a severe threat to the aquaculture environment causing chronic toxicity to the aquatics [38]. Therefore, exposure to sublethal dosages of these metals alarms and stimulates the fishes’ innate immune defense mechanisms differently. In an earlier study, the exposure to sublethal dosages from waterborne heavy metals elicited gilthead seabream (*Sparus aurata*) innate immunity. It increases mucus secretion from the skin, the most directly contacted organ with heavy metals [39]. Although some fish can detoxify Hg^2+^ after some exposure, others do not; this varies according to the fish species, developmental stage, and exposure dosage [40, 41]. The antioxidant activity of the large yellow croaker (*Pseudosciaena crocea*) juveniles was reported to be significantly elevated by their exposure to MeHg (4.0 µg L^−1^) for 30 days; this indicates the high antioxidant defense and innate immunity gained against the heavy metal contamination at the early life stages [42]. In an earlier study, Hg^2+^ toxicity was alleviated from the brain of peacock blennies fish ten days after exposure to a sublethal dose [43]. In contrast, Hg^2+^ was reported to induce chromosomal damage in wild fish (*Dicentrarchus labrax* L.), which doubled and continued this environmental disease for several contrasting seasons [44].

### Feed Additive and Growth Performance

A nominal increase was observed in fish FW in the Hg^2+^ only group, possibly due to a hormetic effect, where a low toxicant dose could have a stimulatory effect [45]. However, the growth-suppressive effect of Hg^2+^ exposure was noticeable, where the feed additive *N. oculata* could not overcome the Hg^2+^ adverse effect. Still, at the same time, it was able to maintain growth within the level of the control one. Our results coincided with previous studies showing a direct relation between Hg^2+^ toxicity and fish growth reduction [46], which might owe to thyroid dysfunction, closely related to metabolism regulation and growth [38, 47]. Notably, the high protein content of *N. oculata* [48] added to feed in our experiment significantly enhanced the healthy fish’s growth (*p* < 0.05). It is known that proteins in tilapia feed improve their growth while increasing their nitrogenous wastes [49]. Noteworthy, the dietary *N. oculata* decreased the HSI without significance (*p* > 0.05) compared with the G_0_ group, indicating no adverse effect on fish health.

### Mercury (Hg^2+^) Bioaccumulation

Our results revealed significantly (*p* < 0.05) higher levels of Hg^2+^ bioaccumulation in the muscles of all Hg^2+^ exposed groups, with a noticeable decrease in its level in the *N. oculata*-supplemented groups compared to the Hg^2+^ group. In addition, traces (0.04 mg/kg) were detected in the muscles of the unexposed fish; such a case is logical since Hg2 + can be created by the metabolism of the vapor of Hg0 or MeHg found naturally by small environmental levels [50]. Bioaccumulation of the Hg^2+^ is promoted by its absorption in biological systems after the deficiency of their antioxidant defense system [18]. In an earlier study, several routes (intraperitoneal injection, oral intubation, or semi-statical exposure) of short-term Hg^2+^ exposure at increasing doses caused dose-dependent Hg^2+^ accumulations and lesions of different severities in tilapia’s (*Oreochromis niloticus*) kidney, hepatopancreas, spleen, and intestine [24]. The relatively low Hg^2+^ concentration in muscle may be due to lower binding affinity with muscle proteins [51]. Similar findings were reported in other fish species exposed to HgCl_2_, including spotted snakehead (*Channa punctata*) [52], Japanese flounder (*Paralichthys olivaceus*) [53], juvenile zebra seabream (*Diplodus cervinus*, Lowe 1838) [54], and catfish (*Heteropneustes fossilis*) [23]. Noteworthy, because of its high content of phenolic and flavonoid compounds, *N. oculata* has a potential chelating effect on Hg^2+^ [55]. These compounds also have a variety of other beneficial biological properties, such as antioxidant and free radical scavenging activities. [56, 57]. Similarly, rohu (*Labeo rohita*) exposed to 0.12 mg/L Hg^2+^ for 21 days had their Hg^2+^ content reduced when fed a diet containing 2% *Spirulina platensis* (*S. platensis*) [30]. When applied at 7 and 10 percent, *Amphora coffeaeformis* reduced arsenic concentration in the muscle of African catfish exposed to arsenic at 38.3 mg/L for 15 days [58]. These findings suggest the potential role of *N. oculata* supplementation as a chelator of toxic metals in fish.

### Serum Acetylcholinesterase (AChE) Activity

Acetylcholinesterase (AChE) activity is considered a valuable tool for recognizing frequent and continuous toxicant exposure [31]. AChE is an enzyme that controls and regulates the transmission of nerve impulses within the cholinergic synapsis; the higher the toxicity and metal ions bioaccumulation, the lower its enzymatic response [5]. Mainly, it catalyzes the hydrolysis process of the “acetylcholine” neurotransmitter into “choline” and “acetic acid,” which is a termination step after the activation prerequisite for neuronal transmission [59]. Our findings showed an improvement in the AChE activity of *N. oculata* dietary supplemented fish, more obviously in the *G*_N10+Hg_, after the significant decrease of its value elicited in the Hg^2+^ only group, which is an indicative factor for the detoxification efficacy of dietary *N. oculata* against the heavy metals’ bioaccumulation. Consistently, three carps, *Labeo rohita*, *Catla*, and *Cirrhinus mrigala*, revealed inhibition of the AChE in a dose-dependent manner when exposed to different concentrations of cadmium, zinc, and their combination [60]. Nile tilapia exposed to diazinon for 45 and 90 days revealed a significant decline in serum AChE that was slightly reversed by bentonite dietary supplementation [61].

### Gene Expression Analysis

The Hg^2+^-only group in gills and intestines exhibited downregulation and upregulation of the relative expression of the targeted antioxidant enzyme-related genes (GSR and GPx) and the targeted stress-related genes (HSP and CAS), respectively. These findings are evident histopathologically, suggesting the harmful impact of Hg^2+^ bioaccumulation in fish tissues that reduces the production of the antioxidant enzyme, which leads to the persistence of oxidative stress. The primary detoxifying function of GPx and GSR is to stop the chain of radicals from propagating, preventing membrane oxidation and damage [62]. In our study, the downregulation of GPx and GSR transcripts during Hg^2+^ exposure could be due to increased utilization of oxidative enzymes for scavenging the H_2_O_2_ and hydroperoxide [63] or as a result of the direct impact of metal ions on the active motifs of the enzyme [64] or the exposed duration [65]. Our results are consistent with previous studies that frequently reported oxidative stress by multiple heavy metals exposure/bioaccumulation. Recently, Alam and his group conducted a study that reported sub-chronic toxicity to the Nile tilapia by dietary MeHg for 60 days, which alters the fish antioxidant status and downregulated the mRNA expression of GSR and GPx genes in the liver [46]. In a similar context, Hg^2+^ toxicity of HgCl_2_-exposed Nile tilapia under thermal stress conditions was marked by a reduction of the antioxidant enzymes, including GPx, in the serum, while a remarkable upregulation of the HSP-encoding gene was noticed in the lateral muscles [63]. More recently, arsenic (As) heavy metal exposure elevated the expression of stress-relevant genes, including HSP, in rohu carp fries [66].

On the contrary, the oxidative stress caused by exposure to MeHg for 30 days significantly elevated the GPx enzyme’s activity in juveniles’ large yellow croaker (Pseudosciaena crocea) [42]. Moreover, a dose-dependent upregulation was reported after Cr^6+^ exposure in expressing several oxidative stress-relevant genes, including the GSR gene, in the liver, owing to oxidative damage [67]. It is worth mentioning that the oxidative stress biomarkers and the degree of oxidative damage in different fish tissues might differ per the tissue of concern, fish age and sex, the dose of exposure, and the thermal conditions [63, 68].

The HSP proteins (HSPs) are essential for protein folding and safeguarding cells from stress; their production is linked to thermal stress caused by high temperatures and metals like cadmium, copper, and mercury [69]. The upregulation of HSP70 in this study by Hg^2+^ exposure in gills and intestines indicates its usefulness as a metal toxicity biomarker. In line with our findings, the juvenile large yellow croaker exhibited upregulation in the expression of HSP70 and HSP27 mRNA exposed to MeHg [42]. The upregulation of four heat shock proteins (HSP22, HSP90 beta, HSP90, and HSP70) was demonstrated in the transcriptomic analysis of Atlantic cod [70] and the liver of Nile tilapia during chronic exposure to chlorpyrifos (CPF) [32]. Similarly, heat stress and Hg^2+^ exposure can upregulate the expression levels of HSP70 in the muscle of Nile tilapia after 21 and 42 days of exposure [63].

To maintain equilibrium in living organisms, a family of conserved intracellular cysteine aspartate-specific proteases known as caspases are responsible for cell regulatory networks controlling inflammation and cell death [71]. In mammals, there are 14 molecules of the caspase protein family: caspases 1–3, 6–9, 12, and 14, which are found in both humans and mice, as well as caspases 4, 5, and 10 in humans (caspase 11 is found in mice) [72]. Initiator caspases (caspase-8 and -9) and executioner caspases (caspase-3, -6, and -7) are the two subclasses of caspases that are involved in apoptosis [71, 72]. Our study revealed an upregulation of the gills and intestines caspase3 mRNA level in the *G*_Hg_ and *G*_N5+Hg_; meanwhile, the *G*_N10+Hg_ succeeded in maintaining its level to the control one. These results coincided with studies that demonstrated the effect of heavy metals on caspasce3 level, including fish *Channa punctatus* exposure to As or Hg [73] or rainbow trout gill-W1 cell lines [74] and testis of *Gobius niger* exposed to Cd [75].

Noteworthy, throughout our study, dietary *N. oculata* elevated the relative expression of the antioxidant enzymes-related genes and suppressed that of the stress protein-related genes. The 10% supplementing dose was more effective. This finding supports the efficacy of *N. oculata* feed supplementation for therapeutic applications against heavy metal contamination. *N. oculata’s* beneficial effects herein could be attributed to its biological constituents, as mentioned earlier, which play a crucial role in different aspects, one aspect as a metal chelator due to its phenolics and flavonoids [55, 76] and antioxidant components [56, 57]. Additionally, the surfaces of algal cells possess several functional groups, including hydroxyl, phosphoryl, amino, carboxyl, and sulphydryl (-SH) groups acting as adsorption sites responsible for heavy metals uptake. The other aspects as an immunomodulator owing to its high content of n3-long chain (LCPUFAs), mainly eicosapentaenoic acid (EPA) and docosahexaenoic acid (DHA), influencing the membrane fluidity of fish cells [77], lead to diminishing the inflammatory changes and augmenting the fish immune defense during [78–80].

In a similar context, several natural products, including algae, were incorporated into the fish diet and reported for protection against the toxicity of heavy metals, including mercury. Increased expression of hepatic and intestinal GST and GPx transcripts in Nile tilapia exposed to air stress has been consistently shown in studies using dietary microalgae like *N. oculata* [81]. During arsenic toxicity, feeding rainbow trout a diet containing *Haematococcus pluvialis* (*H. pluvialis*) extract improved cellular antioxidant defense by upregulating the expression of GPx, SOD, C, AT, and GSTA [82]. The hepatic antioxidative parameters SOD, GSH-Px, and GST gene levels were significantly reduced when *Spirulina platensis* was added to the diet, compared to the sodium sulfate intoxicated group [83]. Zahran et al. [32, 81] and Elabd et al. [84] showed that dietary supplementation with different microalgae positively upregulated the expression of the HSP70 gene in different organs, indicating the immunological properties of phyto-therapies. Moreover, *N. oculata* supplementation was able to diminish the caspase expression level to the control one, particularly in the *G*_N10+Hg_, which is consistent with Singh and his group demonstrating the potential role of ascorbic acid in lowering the caspase expression level in fish *Channa punctatus* exposed to As or Hg [85]. The same results were demonstrated in golden pompano fed on *Odontella aurita* microalga [86].

### Histopathology

Our histopathological findings confirm the protective effect of an *N. oculata*-supplemented diet and support the results of the earlier analyzed biomarkers in the present study. Gills are highly affected as it is the most exposed organ to metal through continuous contact with water [87]. Marked pathological changes were observed in gills and intestinal sections exposed to Hg^2+^, represented by a lamellar fusion and inflammation, leading to a disturbance in gas exchange and decreased fish resistance. Our findings are similar to Nile tilapia gills damage following exposure to 0.03 mg/L HgCl_2_ [88] and Walking catfish (*C. batrachus*) intoxicated with 0.19 mg/L HgCl_2_ for 30 days [89]. The intestine represents a significant route of entry for a wide variety of toxicants present in the diet or in the water that the fish inhabit. In the same trend, the intestine of the exposed fish in *G*_Hg_ showed marked intestinal damage and inflammatory changes that lead to decreased absorptive capability, which are consistent with previous studies on seabream (*Sparus aurata*) and seabass (*Dicentrarchus labrax*) inhabiting the Bardawil Lagoon and exposed to different heavy metals, also on *Channa punctatus* and *Dicentrarchus labrax* exposed to mercury [90, 91], where the intestine showed fragmented, degenerated epithelium, lesions, and disarrangements of mucosal folding.

Interestingly, the gills and intestines of fish groups fed supplemented diets with low or high *N. oculata* exhibited no to mild pathological changes. The complete or partial restoration of tissue in these groups reflects the protective and regenerative capability of *N. oculata*. The absence of inflammation in the intestine and gills reflects the anti-inflammatory and antioxidant effect of *N. oculata*. These results are agreed with our previous studies that showed feeding *N. oculata* reduces the histopathology of Hg^2+^ on the liver, kidney, and gills and has an anti-inflammatory effect on Nile tilapia intestine and liver [26, 81].

## Conclusion

In conclusion, mercuric intoxication in Nile tilapia leads to its accumulation in the musculature, inhibits acetylcholinesterase activity, downregulates genes related to antioxidants and stress, and elicits histopathological changes in the gills and intestine. Dietary supplementation with *N. oculata* effectively mitigated mercury’s harmful effects on fish health. In addition, our research provides strong support for establishing guidelines for the prevention, monitoring, and implementation of a cutting-edge biotech tool for managing aquatic pollution.

## Data Availability

The data supporting this study’s findings are available from the corresponding author upon reasonable request.
